# Progressive structural capacity loss assessment—A framework for modern reinforced concrete buildings

**DOI:** 10.1371/journal.pone.0208149

**Published:** 2018-12-03

**Authors:** Muhammad Zain, Muhammad Usman, Syed H. Farooq, Asad Hanif

**Affiliations:** 1 School of Civil and Environmental Engineering, National University of Sciences and Technology, Islamabad, Pakistan; 2 Department of Civil Engineering, Mirpur University of Sciences and Technology, Mirpur, AJK, Pakistan; Politecnico di Milano, ITALY

## Abstract

By the virtue of burgeoning terrorism, the exponential growth of advanced weaponry, and allied aids for explosions, it is quite evident that infrastructural facilities in the world have increasingly become more susceptible to sabotaging activities. The ever enhancing employment of reinforced concrete (RC) in the construction industry around the globe, the progressive collapse mechanisms, and respective mitigation strategies in the context of terrorism have garnered quite an attraction by the structural engineering community. The proficiency to envisage the complete collapse under the chain reaction of structural failures, partial collapse of key structural members, or the strength degradation of fundamental structural elements under the blast or impact loading can deliver significant information to cope with partial or complete structural failure. It is quite convenient to say that during the service life, a structure may experience extreme loading conditions. The current study has proposed a new methodology to cover the effect of uncertainty involved in loading on key structural elements of new and complex structures by emphasizing a very realistic structural capacity loss mechanism that allows the incremental reduction in the structural capacities of pivotal structural elements against any sort of impact loading instead of their complete annihilation. To demonstrate the application of the proposed methodology, a 13-story complex structure was selected that was comprised of a diverse structural configuration. The outcomes and results ensured the structural integrity against the applied loadings, as well as the effectiveness of the proposed methodology.

## 1. Introduction

The complexities in the structural configuration is none other than a challenge faced by the structural engineering community. The ongoing trend of urbanization tends to break through its conventional records requiring the structural engineering to grow at the same speed. Even with so much improvements in the approaches used for analysis, there still are some gaps that need to be filled for an efficient application of the structural engineering. Several factors like the deficiency of national standards, faulty practices of construction, application of approximate methods of analysis and design of buildings, and inappropriate quality control can lead to the poor quality of concrete constructions [1], and analogously, the improper design of structural members renders the inappropriate re-distribution of gravity loads. Albeit there are some of the studies for structural strengthening e.g. H. Farooq et al. [2]; but previously described adverse mechanisms can triggers the failure of certain portions of a structure which can adopt the shape of a progressive failure or collapse of the structure [3], and large deformations and versatile properties of materials of structural elements different from those exhibited under normal static loading conditions are important to be considered in numerical and analytical modeling of the structures [4].

Keeping in view the mentioned arguments, the current study opens a very new engineering regime towards the challenging professional practice that is focused towards the progressive collapse assessment and proposes a new methodology which reduces the structural strength of elements instead of their complete removal. The current research emphasizes that its quite difficult for an engineered structure to completely loss a structural component in the event of an impact loading or a sabotage activity, rather the stiffness and the strength of component(s) would become significantly diminished under such events. The developed framework considers this slender potential of structural members in reinforced concrete (RC) structures, and barely starts from the selection of the key structural elements in a structure, while giving attention to their sizes, materials’ strengths, and confinements. Subsequently, the developed methodology is focused to reduce the stiffness and load carrying capacity of key structural elements in abrupt increments to evaluate the structural safety, as well as the redundancy to obtain a clear insight of how a structure would perform during any event that can trigger such a situation.

Conventionally, the General Services Administration (GSA) Progressive Collapse Analysis and Design Guidelines (2003) [5] and other official guidelines are employed to evaluate such collapse mechanisms. The main purpose of progressive collapse evaluation is to assess the ability of structural integrity and redundancy to relocate and redistribute the applied loads succeeding the loss or substantial damage of a key load carrying element. Several researchers like Smilowitz [6], Smith [7], and many others utilized GSA guidelines and Department of Defense (DOD) guidelines for executing the PCA. Somayyeh et al. [8] executed the progressive collapse analysis for low-rise moment resisting frames with different eccentricities in plan by completely removing the vertical members, Sagiroglu and Sasani [9] evaluated the potential for progressive collapse resistance of structures following initial damage by starting the removal of columns from the uppermost story and concluded that the ignorance of torsional cracking in beams can lead to a substantial overestimating of the resistance against progressive collapse.

Sasani et al. [10] investigated the progressive collapse resistance of an actual 11 story structure following severe initial damage, analytically and experimentally, which was caused by concurrent explosion of first floor neighboring columns and studied the axial compressive forces in beams. Tsai and Shyu [11] evaluated the dynamic increase factor, ratio of maximum to neutral response, by progressive collapse assessment of a moment resisting frame. Kim et al. [12] investigated the capability of steel moment resisting frame against progressive collapse by employing vertical push-down analysis, and concluded that push-down analysis might overestimate the intrinsic capability of structures to resist progressive collapse. Salloum et al. [13] presented a method to identify acute potential areas which can undergo attack, and the areas subjected to higher stresses, by means of local column analysis and global analysis of the considered 28 story RC tower, and eventually implied that depending upon the obtained information, an effective mitigation plan could be devised, which may be targeted to enhance the redundancy, strengthening of critical columns, and apposite structural detailing for improving ductility of structural connections. Yang et al. [14] presented and elucidated an analytical framework for a probabilistic approach to execute progressive collapse analysis of steel-concrete composite floor using component-based connection model, and eventually compared the attained results with those obtained from deterministic approach.

Liu et al. [15] performed progressive collapse analysis of steel frames under a sudden column removal, and introduced a bearing-capacity index to quantify the robustness of steel frames. Their research concluded that the location, from which the column was removed, and the typology of steel frame i.e. strong-column weak-beam or vice versa, were pivotal to influence the strength of steel frames. Hamid and Majid [16] employed alternate load path method for progressive collapse analysis of 4 story, 8 story, and 15 story steel frames under seismic loading and evaluated the collapse using robustness index, dependent upon stiffness and base shear. Their research concluded that Energy method depicted superior capability for comprehending the structural behavior against seismic loads in progressive collapse scenario.

Yousef et al. [17] assessed the response of precast RC buildings, containing bolted steel plates for precast beam-column connection, in case of sudden column removal. Their research simulated the scenario by removing a central column, and evaluated the structural response under different steel plate parameters. However, scarcity exists in the studies related to precast structures. Nascent developments i.e Cong Lu et al. [18], Daniel de Lima et al. [19], etc. must also be evaluated against progressive collapses and other impact loads. Feng and Michel [20] performed reliability analysis to assess the redundancy of a box-girder bridge and a truss bridge. Their research implied that despite of the broader application of GSA Guidelines in the design of buildings, they cannot be conveniently applied for the bridges because of the differences in the nature of their structural behaviors, and the transient loads that act upon them. The research proposed a probabilistic dynamic progressive collapse analysis methodology and a calibrating incremental analysis criterion to incorporate the uncertainties and the variations in load carrying capabilities of the members and the applied loads. Setareh et al. [21] studied the effects of structural capacities on Dynamic Increase Factor (DIF), a factor used to account for dynamic effects when linear static and nonlinear static methods are employed for progressive collapse analysis, in RC structures, and eventually proposed a new empirical DIF formula by analyzing numerous three-dimensional RC frames with different number of stories and span lengths, with varying levels of seismic resistances, and concluded that the proposed formula would be helpful in determining the stresses and deformations in RC structures after the removal of a column. Yu and Tan [22] conducted experimental program to assess the resistance of reinforced beam-column sub-assemblages under the removal of a middle column against the progressive structural failure by using alternate load path method and concluded that relative to conventional yielding strength, compressive arch actions and catenary action substantially increases the structural capacity against progressive collapse.

Contrary to conventional progressive collapse analysis approaches, the proposed procedure does not allow to remove the structural element completely, in fact, it allows the incremental reduction in the capacity of the key structural element(s) up to the extent decided and considered by the engineer. This reduction depends upon the Force-Deformation (F-D) Relationships of the considered elements which can also take into account the effect of concrete confinements. The proceeding headings and paragraphs presents the newly established framework and considers a case study to elaborate the practical application of the presented methodology.

## 2. Method and materials

**Fig 1:**
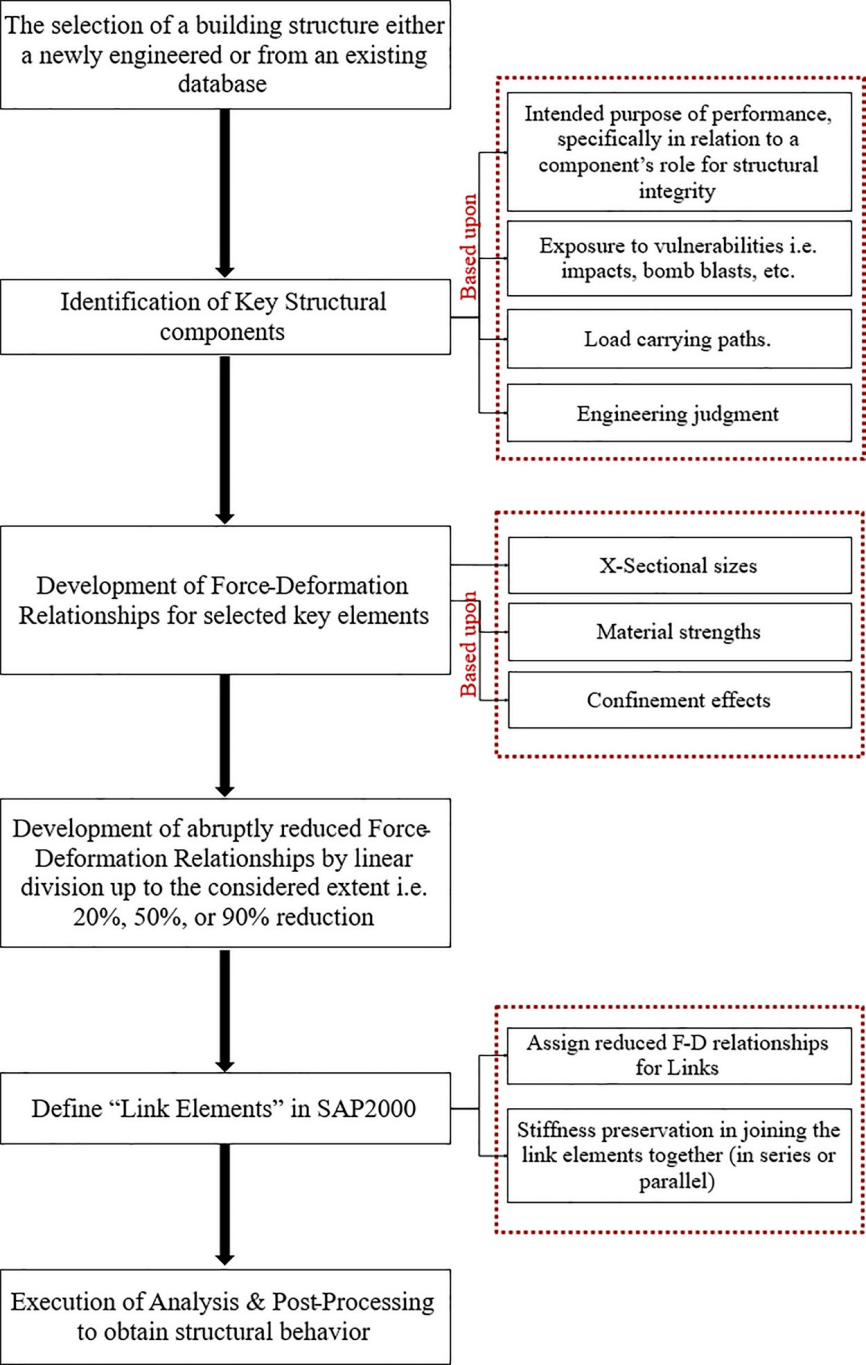
Proposed methodology for “progressive structural capacity loss assessment”.

The method utilizes nonlinear static analysis technique to capture the nonlinearity involved. Fig 1 shows the hierarchy of the proposed methodology in the form of a flow chart. The figure itself clearly explains the technique used for the execution of analysis.

A 13 story mid-rise building, located in Brunei Darussalam was selected to demonstrate the execution of the proposed methodology. A 3D nonlinear analytical model was created in CSI SAP2000, as the software is well capable to pursue the required nonlinear analysis which demands the usage of special elements whose F-D relationships can be controlled [23, 24], thus, the link elements provided in the software, exactly match with the precise requirement, and at the same time, the software allows the load to be locally redistributed in cases when the element is not considered to be carrying the applied gravity loads, properly.

### 2.1 Analytical modelling and geometry of structure

**Fig 2:**
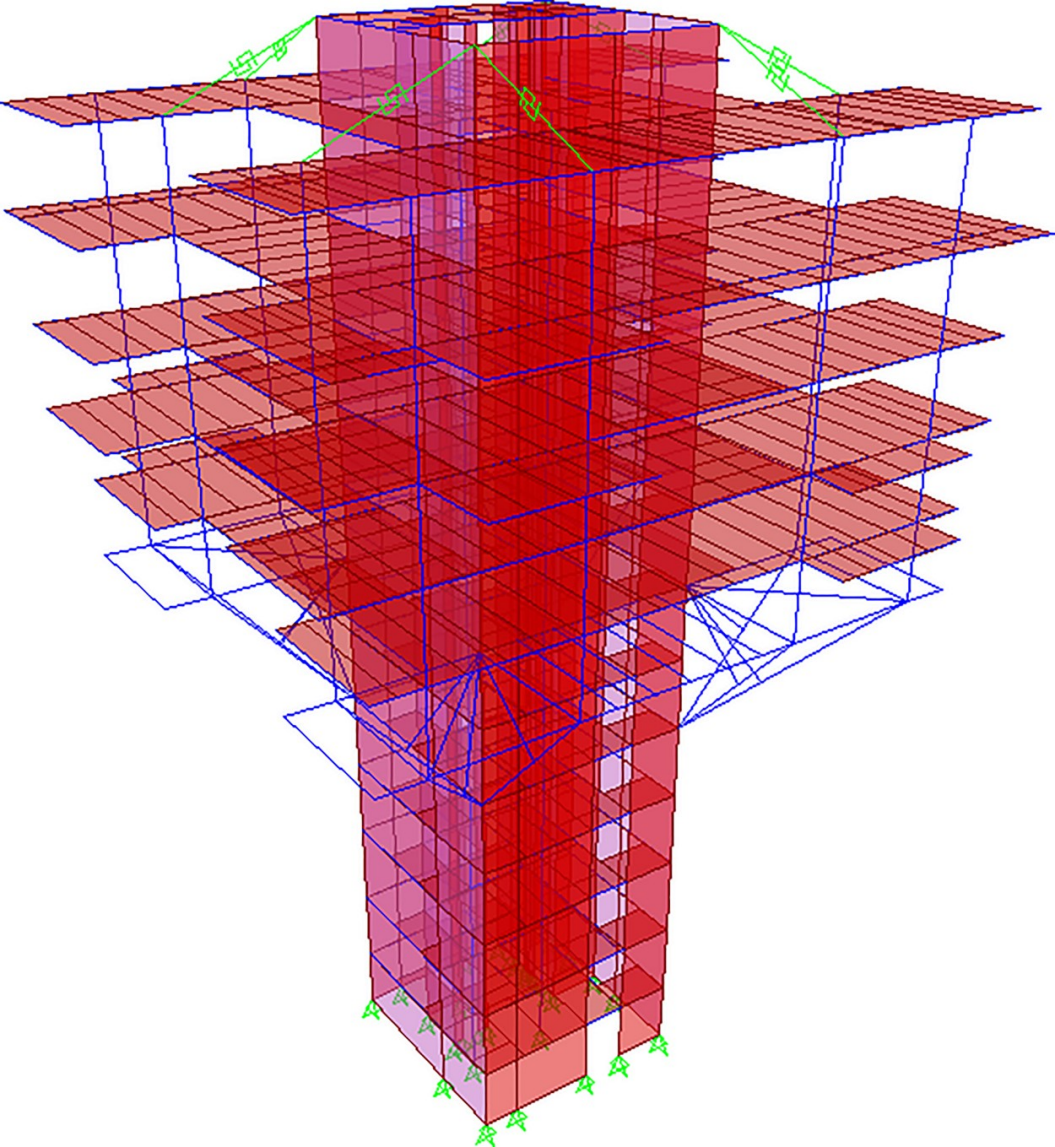
3D Analytical model of the considered building, showing the propagation and projection of diagonal struts from 5^th^ floor to 7^th^ floor, as well as the ties at the top. Central diagonal strut at each side of the structure consisted of 13 meters of length, while the two, side diagonal struts, at each corner of the structure, comprised of 10.8 meters of length. Each strut possessed a typical cross-section of 800mm X 800mm, and a yielding capacity of 25548 KN (the yielding capacity was evaluated using actually expected material strengths).

**Fig 3:**
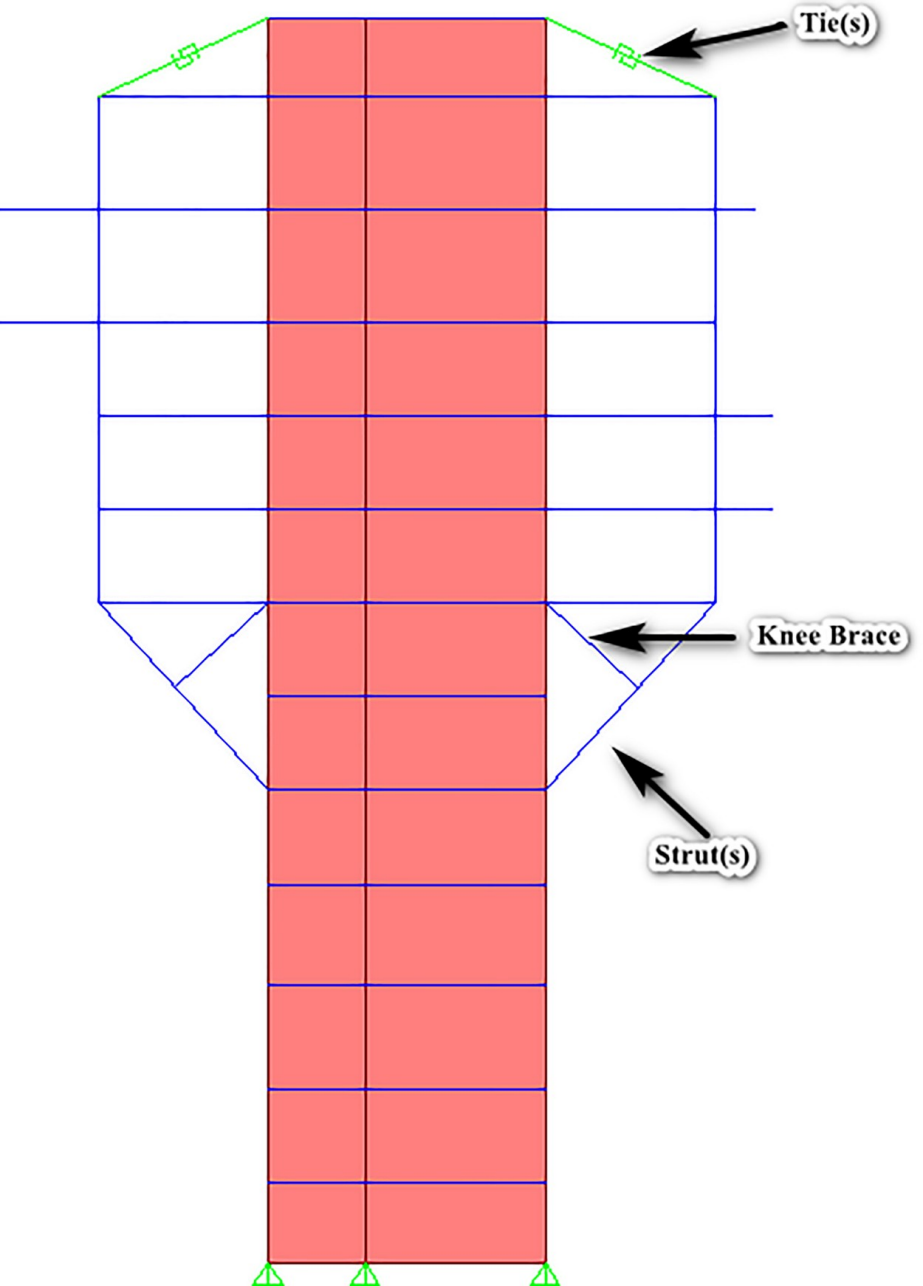
Elevation view showing the specific locations of strut(s) and tie(s). To produce the inherent redundancy in the structure, steel ties were provided at the roof deck level to ensure that the structure remains standing and no loss of life occurs even when any or all of the struts lose their capacity up to significant level. The ties were modelled as multi-linear plastic link elements as link elements can be utilized to model special behavior (CSI, 2011). The ties conceptually tolerated the load only when the struts were supposed to fail, and they were essentially modelled to remain in tension when the struts lose their respective capacities. The F-D relationships of steel ties, the link elements, were primarily dependent upon the material used for their fabrication; Fig 4 shows the F-D relationship evaluated for ties.

The geometry of the structure consisted of a straight and rectangular core wall up to the roof deck, but from the 5^th^ floor, 12 diagonal struts were projected outwards (three struts from each corner of the structure) which started propagation from 5^th^ floor and diagonally ended it at the 7^th^ floor, as can be observed in Fig 2, and Fig 3 as well. Fig 2 shows the complete 3D nonlinear analytical model, made in SAP2000. The struts were modeled as frame elements. A “knee brace” frame element was also provided in the structure to support the diagonal struts, as can be seen in Fig 2. For the actual visualization of these members, Fig 3 shows the elevation view of the structure that elaborates the members in a more sophisticated manner.

**Fig 4:**
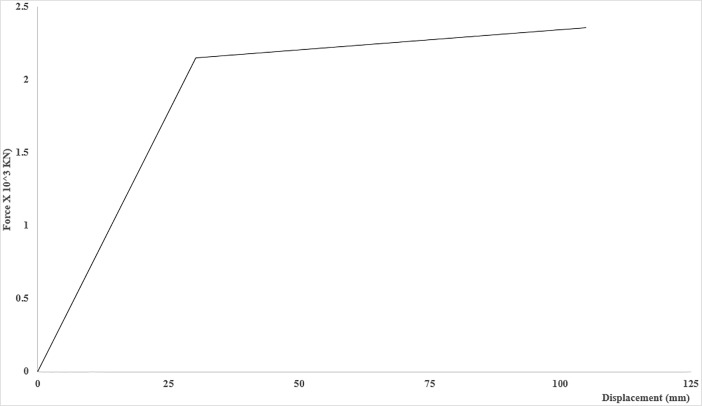
Force-Deformation Relationship of Steel Ties, located at the roof deck level. As described; the procedure for progressive structural capacity loss assessment is uniquely based on incremental reduction of structural strength, thus, a scenario based Progressive Structural Capacity Loss Assessment (PSCLA) was performed. The nonlinearity in the model was introduced by means of concentrated hinges at certain critical elements.

### 2.2. Scenarios for PSCLA

Specific scenarios of capacity loss of key structural elements were considered as described under this heading. The diagonal strut members were considered to be the most critical key structural elements in the building and were targeted to execute the PSCLA. For performing PSCLA, four specific scenarios were decided as mentioned:

- When one (central) diagonal strut of a side lost its capacity
- When two (Side) diagonal struts lost their capacities
- When all three diagonal struts of a side lost their capacities
- When every diagonal strut of every side lost its capacity

The structural geometry of the selected building allowed a particular side of the tower to have more load than the others, therefore, the struts comprising that portion were considered for the first three scenarios for simulating the *worst-case* conditions.

The capacity loss of struts itself comprised upon two consecutive stages. In the first stage, the elements lost their capacities by 50% (50% capacity remaining), while in the second stage, the elements lost their capacities by 75% (25% capacity remaining).

PSCLA is based upon the *“Performance-Based Assessment”* approach, thus, the expected values of material strengths were used in the analysis, and a complete *non-linear static analysis* was performed to ensure the structural integrity and its safety in opposition to the application of loads. Complete and proper application of gravity load (dead, live, and superimposed dead loads) was accurately considered during the analysis.

### 2.3. Procedure for PSCLA

The struts were dealt as axial-compression members. Initially, their axial stiffness was calculated, and based upon their stiffness values and material strengths, précised force-deformation relationships were calculated to define their yielding and ultimate strengths. Link Elements were then used to replace the frame (struts) elements in the analytical model. The modeling of these elements are addressed in proceeding section.

### 2.4. Replacing link elements for struts

Link elements, upon the calculated F-D relationship, were then modeled in SAP 2000, and for each scenario of capacity reduction, the particular strut(s) were replaced by specific link elements. All the link elements were modeled as multi-linear plastic elements using “Multi-linear Takeda Plasticity Property”, the reader is referred to [2] for details on multi-linear takeda plasticity property. Table 1 contains the information on modelling parameters.

**Table 1:** Modelling parameters for diagonal struts and link elements.

Modelling Parameters	Symbol	Equation
Axial Capacity in Compression Phase (Using expected strengths)	N_C_	0.85 × *f_c_*′ × (*A_g_* − *A_st_*) + *A_st_f_y_*
Axial Stiffness	K	$\frac{EA}{L}$
Yield Displacement in Compression	D_yc_	$\frac{N}{K}$
Axial Capacity in Tension Phase	N_T_	*f_y_* × *A_st_*
Yield Displacement in Tension	D_yt_	*ε_st_* × *L_m_*

Where

f_c_’ is 28 days concrete cylindrical strength

A_g_ is gross area of X-section

A_st_ is area of steel reinforcements

E is Young’s Modulus

F_y_ is yielding stress of reinforcing bars

ε_st_ is strain in reinforcing steel

L_m_ is length of the element

The ultimate displacements were dependent upon the material stress-strain curves which were eventually converted into Force-Deformation Relationships for each of the considered element. For each strut, two link elements were used, as the software does not allow an automatic connection at intermediate points between link and frame elements. The first one ended at the knee brace, and the second link element was started from the knee brace.

The link elements were joined in series, but same stiffness was preserved as that of actual strut member, in each scenario of capacity loss. As described, the link elements were joined in series, the stiffness calculations were based upon the following equation.

$$\frac{1}{K_{STRUT}}=\frac{1}{K_{LINK1}}+\frac{1}{K_{LINK2}}$$ (1)

**Fig 5:**
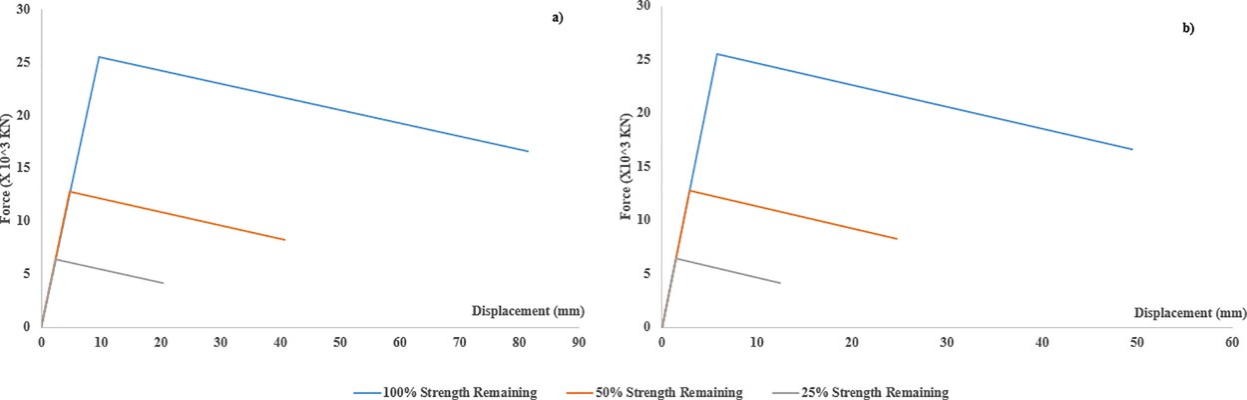
a) F-D relationships of replacing link element, 8.14 m in length b) F-D relationships of replacing link element, 4.96 m in length (spring elements F-D relationships for 13m long strut).

**Fig 6:**
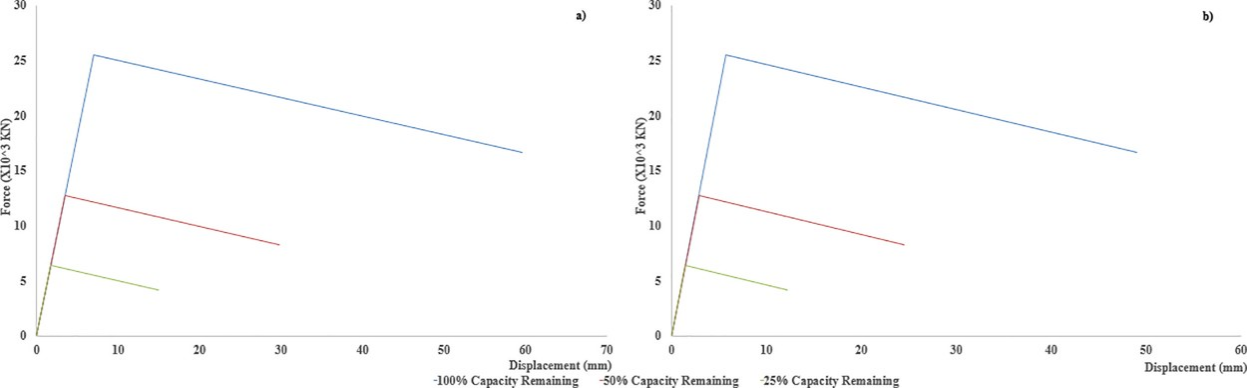
a) F-D relationships of replacing link element, 5.96 m in length b) F-D relationships of replacing link element, 4.9 m in length (spring elements F-D relationships for 10.8 m long strut).

Where K_STRUT_ represents the stiffness of the actual strut, while the K_LINK1_ and K_LINK2_ describe the respective stiffness of the two links, which were used to replace the actual strut. F-D relationships of two link elements in compression, which replaced the length of 8.14m and 4.96m, separately, of the whole strut of 13m length, are shown in Fig 5(A) and 5(B) respectively, while the F-D relationships for link elements, 5.96m and 4.9m, which replaced the strut of 10.8m of length are given in Fig 6(A) and 6(B) respectively. These figures also elaborate the mechanism of *“strength reduction”* for each consecutive scenario and it is clearly described that in each scenario, the strength was reduced but the stiffness of the element (slope of the curve) remained constant.

## 3. Results and discussions

The results obtained from the analysis are self-explaining about the efficiency of proposed procedure. For checking the adequacy, first analysis simulation was carried out using the struts, intact in their original condition, while all other simulations that contain the scenarios for capacity reduction, were executed using the link elements.

### 3.1. Forces in strut(s) and tie(s)

Table 1 shows the results in terms of forces in the struts and the forces in the ties along with their elongation against the applied loads. First column of Table 2 describes the strut IDs (3 IDs for each corner) in the model while the tie IDs, mentioned in front of each set of strut IDs, represent the ties attached at the roof level, above those particular struts. “Negative” sign with the values show the compression behavior of the elements, while the positive values describe the tension in the elements (tie(s)).

**Table 2:** Forces in struts and ties.

Strut ID	Strut Demand Forces (KN)	Tie ID	Tie Demand Forces (KN)	Elongation in Ties (mm)
128	-3556.01	11	220.07	3.16
275	-4462.45	16	134.80	1.94
641	-3606.54			
131	-4360.56	12	211.91	3.04
236	-3750.26	18	166.20	2.39
296	-3786.03			
254	-3577.46	14	244.97	3.52
215	-4416.97	17	196.76	2.83
299	-3559.84			
212	-4351.08	13	242.07	3.48
257	-3699.82	15	223.85	3.22
278	-3795.47			

After Performing each step of capacity loss in each scenario, the forces in replaced link elements and in each tie were calculated to verify the results. The details can be examined in the proceeding section.

### 3.2. Forces in replacing link element(s) and tie(s)

**Fig 7:**
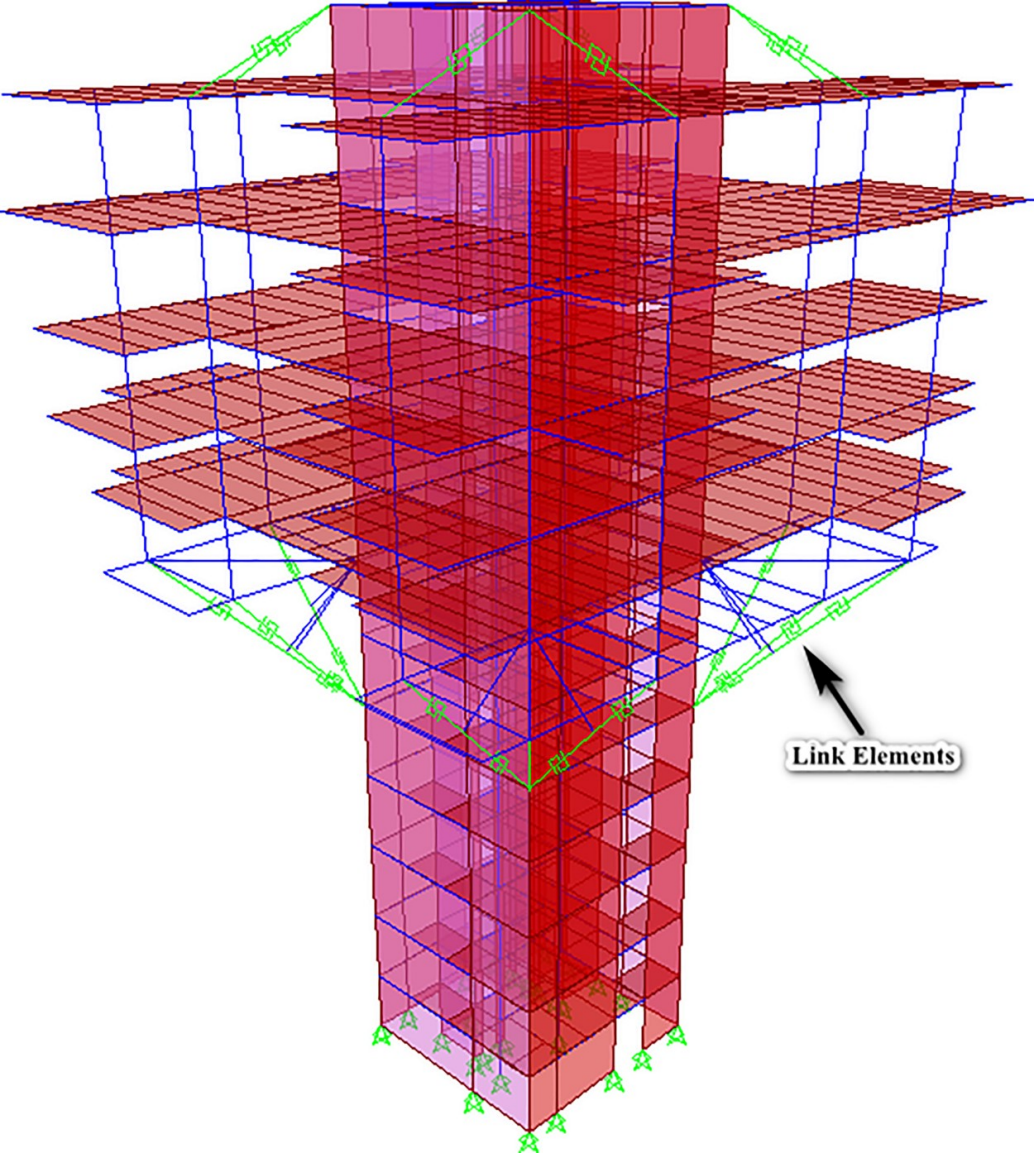
3D Analytical model after replacing the struts with link elements.

**Fig 8:**
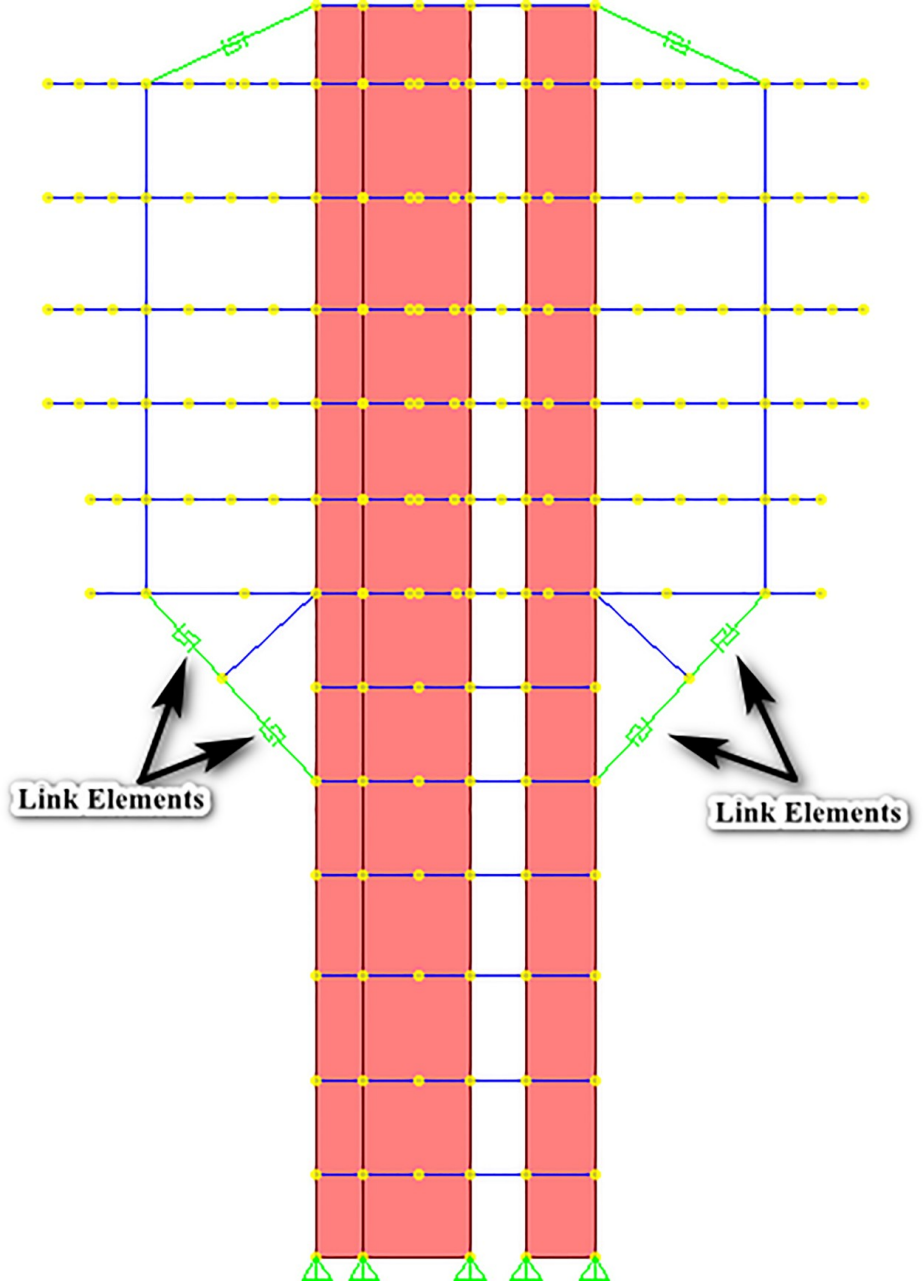
3D Analytical model after replacing the struts with link elements.

Fig 7 and Fig 8 show the full 3D analytical model and an elevation view after the assignments of respective link elements in place of struts, respectively. The Figs 7 and 8 elaborate the case of 4^th^ scenario, in which all struts of structure were replaced and were considered to be in the condition where they had lost their capacities up to 50%.The results presented herewith only correspond to 4^th^ scenario as it is considered to be the most vulnerable condition of the structure. The results are provided in tabular form. Table 3 and Table 4 show the forces in the replaced link elements and forces in ties, along with their elongation, when the capacities of all the struts are reduced up to 50% and 75% respectively (the fourth scenario). Similar to Table 2, first column of tables describes the strut IDs (3 IDs for each corner) in the model, While the second column of the tables does not show the forces in struts (as all the struts are replaced by corresponding link elements), instead, the third column of tables describes about the forces in the replacing link elements, that were found to be analogous as in the case of intact struts. Tie IDs, which are mentioned correspond to each set of strut IDs, represent the ties attached at the roof level, above those particular struts. “Negative” sign shows the values in compression, while the positive values show tension behavior of the element.

**Table 3:** Forces in replacing link elements and Ties– 4^th^ scenario– 50% capacity reduced.

Strut ID	Strut Demand Forces (KN)	Replacing Link Element(s) Forces (KN)	Tie ID	Tie Demand Forces (KN)	Elongation in Ties (mm)
128	Replaced	-3555.35	11	220.07	3.16
275	Replaced	-4463.52	16	134.80	1.94
641	Replaced	-3606.54			
131	Replaced	-4360.56	12	211.91	3.04
236	Replaced	-3761.48	18	166.20	2.39
296	Replaced	-3786.33			
254	Replaced	-3578.81	14	244.97	3.52
215	Replaced	-4416.73	17	196.76	2.83
299	Replaced	-3558.14			
212	Replaced	-4358.88	13	242.07	3.48
257	Replaced	-3693.62	15	223.85	3.22
278	Replaced	-3798.63			

**Table 4:** Forces in replacing link elements and ties– 4^th^ scenario– 75% capacity reduced.

Strut ID	Strut Demand Forces (KN)	Replacing Link Element(s) Forces (KN)	Tie ID	Tie Demand Forces (KN)	Elongation in Ties (mm)
128	Replaced	-3555.35	11	220.07	3.16
275	Replaced	-4463.52	16	134.80	1.94
641	Replaced	-3606.54			
131	Replaced	-4360.56	12	211.91	3.04
236	Replaced	-3761.48	18	166.20	2.39
296	Replaced	-3786.33			
254	Replaced	-3578.81	14	244.97	3.52
215	Replaced	-4416.73	17	196.76	2.83
299	Replaced	-3558.14			
212	Replaced	-4358.88	13	242.07	3.48
257	Replaced	-3693.62	15	223.85	3.22
278	Replaced	-3798.63			

## 4. Conclusions

As evident from the results, there was not much considerable difference in the forces of replaced link elements and ties. (comparison of Table 2 with Table 3 and Table 4), as the yielding capacity of a single strut comes out to be 25548 KN, but the maximum demand force is only up to 4462 KN. Conclusively, If a strut losses its capacity by 75% (which comes out to be 19161 KN; and portrays the yielding capacity as 6387 KN), the struts and ties, both of them will not be yielding, and results elaborated the same (Tables 3 and 4), providing verification to the conception. It is quite difficult to compare the results with some other results obtained from conventional progressive collapse analysis as the presented framework is essentially a one of its kind till date as instead of a complete removal of a structural element it substantially focusses on reducing the structural capacities. However, the results are self-explanatory and defend themselves when the obtained forces in link elements are compared and found analogous with those of actual struts when they were intact before replacing with link elements that contain derived F-D relationships and stiffness to represent the actual struts.

The proposed methodology has provided more rational and practical approach towards the capacity loss assessment. It was not focused towards the complete removal of the element(s), rather it allowed the incremental reduction of the capacity itself. At the same time, the proposed procedure allowed the preservation of same stiffness even when the capacity was abruptly reduced, as can be imagined in case of a bomb blast, collision or some other type of impact loading, and turned out to be more realistic and rational approach towards the engineering practices.

## Supporting information

S1 Fig Proposed methodology for “progressive structural capacity loss assessment. (XLSX)

S2 Fig 3D analytical model of the considered building, showing the propagation and projection of diagonal struts from 5^th^ floor to 7^th^ floor, as well as the ties at the top. (XLSX)

S3 Fig Elevation view showing the specific locations of strut(s) and tie(s). (XLSX)

S4 Fig Force-deformation relationship of steel ties, located at the roof deck level. (XLSX)

S5 Fig a) F-D relationships of replacing link element, 8.14 m in length b) F-D relationships of replacing link element, 4.96 m in length (spring elements F-D relationships for 13m long strut).
(XLSX)

S6 Fig a) F-D relationships of replacing link element, 5.96 m in length b) F-D relationships of replacing link element, 4.9 m in length (spring elements F-D relationships for 10.8 m long strut).
(XLSX)

S1 Table Modelling parameters for diagonal struts and link elements. (XLSX)

S2 Table Forces in struts and ties. (XLSX)

S3 Table Forces in replacing link elements and ties– 4^th^ scenario– 50% capacity reduced. (XLSX)

S4 Table Forces in replacing link elements and ties– 4^th^ scenario– 75% capacity reduced. (XLSX)
